# Parathyroid Hormone-Related Protein Negatively Regulates Tumor Cell Dormancy Genes in a PTHR1/Cyclic AMP-Independent Manner

**DOI:** 10.3389/fendo.2018.00241

**Published:** 2018-05-16

**Authors:** Rachelle W. Johnson, Yao Sun, Patricia W. M. Ho, Audrey S. M. Chan, Jasmine A. Johnson, Nathan J. Pavlos, Natalie A. Sims, T. John Martin

**Affiliations:** ^1^Department of Medicine, Division of Clinical Pharmacology, Vanderbilt Center for Bone Biology, Vanderbilt University Medical Center, Nashville, TN, United States; ^2^Bone Biology and Disease Unit, St. Vincent’s Institute of Medical Research, Fitzroy, VIC, Australia; ^3^Department of Medicine at St. Vincent’s Hospital, University of Melbourne, Melbourne, VIC, Australia; ^4^Cellular Orthopaedic Laboratory, School of Biomedical Sciences, The University of Western Australia, Crawley, WA, Australia

**Keywords:** parathyroid hormone-related protein, cyclic AMP, MCF7, breast cancer, calcium signaling

## Abstract

Parathyroid hormone-related protein (PTHrP) expression in breast cancer is enriched in bone metastases compared to primary tumors. Human MCF7 breast cancer cells “home” to the bones of immune deficient mice following intracardiac inoculation, but do not grow well and stain negatively for Ki67, thus serving as a model of breast cancer dormancy *in vivo*. We have previously shown that PTHrP overexpression in MCF7 cells overcomes this dormant phenotype, causing them to grow as osteolytic deposits, and that PTHrP-overexpressing MCF7 cells showed significantly lower expression of genes associated with dormancy compared to vector controls. Since early work showed a lack of cyclic AMP (cAMP) response to parathyroid hormone (PTH) in MCF7 cells, and cAMP is activated by PTH/PTHrP receptor (PTHR1) signaling, we hypothesized that the effects of PTHrP on dormancy in MCF7 cells occur through non-canonical (i.e., PTHR1/cAMP-independent) signaling. The data presented here demonstrate the lack of cAMP response in MCF7 cells to full length PTHrP(1–141) and PTH(1–34) in a wide range of doses, while maintaining a response to three known activators of adenylyl cyclase: calcitonin, prostaglandin E_2_ (PGE_2_), and forskolin. PTHR1 mRNA was detectable in MCF7 cells and was found in eight other human breast and murine mammary carcinoma cell lines. Although PTHrP overexpression in MCF7 cells changed expression levels of many genes, RNAseq analysis revealed that PTHR1 was unaltered, and only 2/32 previous PTHR1/cAMP responsive genes were significantly upregulated. Instead, PTHrP overexpression in MCF7 cells resulted in significant enrichment of the calcium signaling pathway. We conclude that PTHR1 in MCF7 breast cancer cells is not functionally linked to activation of the cAMP pathway. Gene expression responses to PTHrP overexpression must, therefore, result from autocrine or intracrine actions of PTHrP independent of PTHR1, through signals emanating from other domains within the PTHrP molecule.

## Introduction

Parathyroid hormone-related protein (PTHrP, gene name *PTHLH/Pthlh*) is a cytokine with functions in both pathology and physiology (1, 2). Although it was identified as the circulating factor responsible for humoral hypercalcemia of malignancy (3), it more commonly acts in a paracrine manner: in breast cancer cells it promotes their metastasis (4, 5), in bone (osteoblasts and osteocytes) it stimulates bone formation (6, 7), and in cartilage cells (chondrocytes) it controls proliferation and hypertrophy (8).

In breast cancer cells that lay dormant in bone (9) we have previously shown that overexpression of PTHrP enables otherwise dormant human MCF7 breast cancer cells to aggressively colonize the bone marrow and induce osteolysis (5). Consistent with enhanced bone colonization, we recently reported that such overexpression of PTHrP in MCF7 cells results in the downregulation of several pro-dormancy genes (9).

The best understood actions of PTHrP are those that are mediated by its binding to the G protein-coupled receptor that it shares with parathyroid hormone (PTH) (PTHR1). Upon ligand binding to the receptor, cyclic AMP (cAMP) is activated, followed by protein kinase A (PKA) activation, cAMP responsive element binding protein (CREB) phosphorylation, and transcription of CREB target genes (10–13). This PTHR1-dependent signaling pathway is shared between PTH and PTHrP due to high sequence homology in their amino-terminal domains; the portion of the molecule that interacts with the receptor (14). Early work showed that MCF7 cells failed to respond to PTH treatment with any increase in cAMP or activation of cAMP-dependent protein kinase, suggesting that PTHR1 in those cells is not functionally linked to adenylyl cyclase (15). In contrast, MCF7 cells possess specific, high affinity receptors for calcitonin linked to adenylyl cyclase activation, and activate cAMP in response to prostaglandin E_2_ (15, 16). These data suggest that the effect of PTHrP overexpression on tumor dormancy in MCF7 cells may occur through PTHR1-independent actions of the PTHrP molecule.

Using multiple assays, we report here that MCF7 cells, and many other breast cancer cell lines, express PTHR1 mRNA but do not bind PTH, nor do they activate cAMP formation or subsequent cAMP signaling events in response to PTH or PTHrP. Our RNAseq analyses identify many genes induced by PTHrP overexpression in MCF7 cells, and several potential alternative pathways, notably those related to calcium signaling.

## Materials and Methods

### Cell Culture

Human MCF7 cells were obtained from ATCC and grown in DMEM supplemented with 10% FBS and penicillin/streptomycin (P/S). MCF7pcDNA and MCF7 PTHrP-overexpressing cells were generated as described previously (5) and grown in the same conditions as MCF7 cells; we utilized strains grown and maintained at two separate institutions to validate findings. All breast cancer and mouse mammary carcinoma cell lines were obtained and grown as previously described (9). The rat osteosarcoma (UMR106-01) cell line was maintained in DMEM supplemented with 10% FBS and P/S as described in Ref. (17). MC3T3-E1 cells were maintained in α-MEM supplemented with 10% FBS as described in Ref. (18).

### cAMP Response Assay

Briefly, MCF7 cells were cultured in 12-well plate in cell culture media containing 1 mM isobutylmethylxanthine. Cells were then treated for 12 min with either PTH (100 nM) (sourced from Bachem, Bubendorf, Switzerland), PTHrP(1–141) (100 nM) [expressed in *Escherichia coli* and purified in house (7)], or the known agonists forskolin (10 µM) (sourced from Sigma), prostaglandin E_2_ (1 µM) (sourced from Sigma), or salmon calcitonin (sCT) (1 µM) (kindly gifted by the late Dr. M Azria, Novartis AG, Basel, Switzerland). The cells were washed, acidified ethanol was added, and after air drying was reconstituted in assay buffer and cAMP formation assayed as previously (19).

### CRE-Luciferase Assay

MCF7 cells were transiently transfected with cAMP response element (pCRE)-luciferase (Clontech), a vector containing multiple copies of CRE binding sequences. Fugene (Promega) was used to transfect cells. Four hours after agonist stimulation, cells were lysed, substrate (Promega) was added, and signal was measured using a Polarstar Optima.

### Real-Time Quantitative PCR

Cell lines were harvested in TRIzol (Life Technologies) or TriSure (Bioline) for phenol/chloroform extraction of RNA, DNAse digested (TURBO DNA-free kit, Life Technologies), and cDNA was synthesized from 200 ng–1 µg RNA (iScript cDNA synthesis kit, Bio-Rad or Tetro cDNA synthesis kit, Bioline) per the manufacturer’s instructions as previously described (9). Real-time PCR was performed on either a Quantstudio5 384-well plate format (Thermo Fisher) or Stratagene MX3000P (Agilent) with the following cycling conditions: 2 min at 50°C, 10 min at 95°C, (15 s at 95°C, 1 min at 60°C) × 40 cycles, and dissociation curve (15 s at 95°C, 1 min at 60°C, 15 s at 95°C) or 10 min at 95°C, (30 s at 95°C, 1 min at 60°C) × 40 cycles, and dissociation curve (1 min at 95°C, 30 s at 55°C, 30 s at 95°C). Primers for mouse *PTHR1* were previously published (20) and human *PTHR1* primer sequences were sourced from MGH Primerbank (F: CTGGGCATGATTTACACCGTG, R: CAGTGCAGCCGCCTAAAGTA). Human *PTHLH* primers were previously published (21) and human *HPRT1, RGS2, CREB, PRKAR1, AREG*, and *NR4A1* primers were previously published (22). Primer sequences for human *BDKRB1* and *CALML3* were designed using PrimerBLAST (*BDKRB1* F: AATGCTACGGCCTGTGACAA, R: TCCCTAGGAGGCCGAAGAAA; *CALML3* F: TGGTTGATTCAGCCCACCTC, R: TCCGTGTCATTCAGACGAGC). Gene expression between samples was normalized to *B2M* expression or *B2M*: *HPRT1* geometric mean. Relative expression was quantified using the comparative CT method [2^−(Gene Ct–Normalizer Ct)^].

### Confocal Microscopy

#### Antibodies and Reagents

Tetramethylrhodamine (TMR)-labeled PTH(1–34) (PTH^-TMR^) was synthesized as previously described (23). Anti-VPS35 mouse monoclonal was purchased from Santa Cruz Biotechnology Inc., USA. Alexa Fluor 488 anti-mouse secondary antibody was purchased from Molecular Probes^®^, Invitrogen, USA.

#### Imaging

MCF7 and UMR106-01 cells were cultured as described above, and seeded on poly-l-lysine-coated glass coverslips at 1 × 10^4^ cells/well (96-well plate) for 24–48 h prior to agonist stimulation. Cells were then serum starved for 1 h prior to the addition of PTH^-TMR^ (100 nM) for 15 min at 37°C. Cells were then washed in ice-cold 1× PBS and fixed in 4% PFA at room temperature, permeabilized with 0.1% Triton X-100 for 5 min, washed in 0.2% BSA-PBS, and blocked in 3% BSA-PBS for 30 min. Cells were then incubated with anti-VPS35 antibody (Santa Cruz Biotechnology Inc.) for 1 h at room temperature, and washed in 0.2% BSA-PBS and 1× PBS prior to incubation with Alexa Fluor 488 anti-mouse secondary antibody (Molecular Probes^®^, Invitrogen), for 45 min at room temperature. Cells were then stained with DAPI stain and mounted in ProLong^®^ Diamond Antifade (Molecular Probes^®^, Invitrogen). Detection of immunofluorescence was performed using a Nikon A1Si confocal microscope running NIS-C Elements Software (Nikon Corp., Japan). A 40× oil immersion objective lens (Nikon, Japan) was used, where serial optical sections (*z*-stack) of 0.5–1 µm were used to reconstruct 2D projections in FIJI (NIH, USA).

### RNA Sequencing and Bioinformatics

RNA samples of MCF7pcDNA control and MCF7 PTHrP-overexpressing cells (*n* = 3 independent replicates/group) were submitted to the Stanford Functional Genomics Facility and analyzed for RNA integrity using a Bioanalyzer (Eukaryote Total RNA Nano, Agilent) and all samples had a RNA integrity number of 9.50–10 (10 is highest quality possible). RNA samples were sequenced on an Illumina NextSeq with coverage of approximately 40 million reads per sample. Sequence alignment and RNAseq bioinformatics analysis was performed by the Vanderbilt Technologies for Advanced Genomics Analysis and Research Design (VANGARD) core at Vanderbilt University Medical Center. RNAseq files are available in the GEO repository (GEO accession number GSE110713).

### Statistics

All data are presented as the mean of *n* = 3 biological replicates obtained from three independent experiments (one biological replicate, with three technical replicates per experiment). For all graphs error bars indicate the SEM. Statistical tests used are indicated in the figure legends, and *p*-values were considered significant at *p* < 0.05.

## Results

### PTHR1 mRNA Is Detected in Breast Cancer Cells

**Figure 1:**
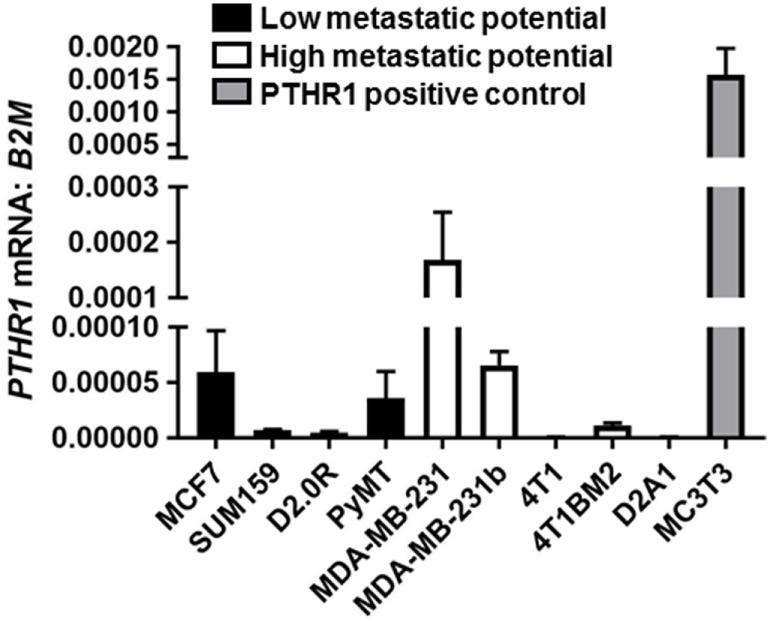
PTHR1 is expressed by breast cancer cells. *PTHR1* mRNA levels in human breast cancer cell lines {MCF7, SUM159, MDA-MB-231, MDA-MB-231b [bone metastatic clone (25, 26)]}, mouse mammary carcinoma cell lines {D2.0R, PyMT, 4T1, 4T1BM2 [bone metastatic clone (27)], D2A1}, classified according to metastatic potential, and PTHR1/cyclic AMP responsive MC3T3-E1 cells. mRNA levels were normalized to β-2-microglobulin (*B2M*) housekeeping gene. Graphs = mean + SE. *n* = 3 replicates from independent experiments.

PTHR1 mRNA levels varied but were detectable across all human breast cancer and mouse mammary carcinoma cell lines tested (Figure 1). The panel included cell lines termed “high metastatic potential” [that aggressively colonize the bone after intracardiac inoculation or lung after tail vein inoculation (9)], and cell lines termed “Low metastatic potential” (9) [that do not colonize, or proliferate very slowly after inoculation (9)]. PTHR1 mRNA levels did not correspond to the metastatic potential of the cell lines. 4T1 and D2A1 cells had the lowest expression of PTHR1, which was nearly undetectable (4T1: Ct values = 33–39; D2A1: Ct values = 33–34). All breast cancer cell lines had at least 10-fold lower *PTHR1* mRNA levels than MC3T3-E1 cells, which have a robust cAMP response to exogenous PTH and PTHrP treatment (24).

### Neither PTH nor PTHrP Stimulates cAMP in Breast Cancer Cells

**Figure 2:**
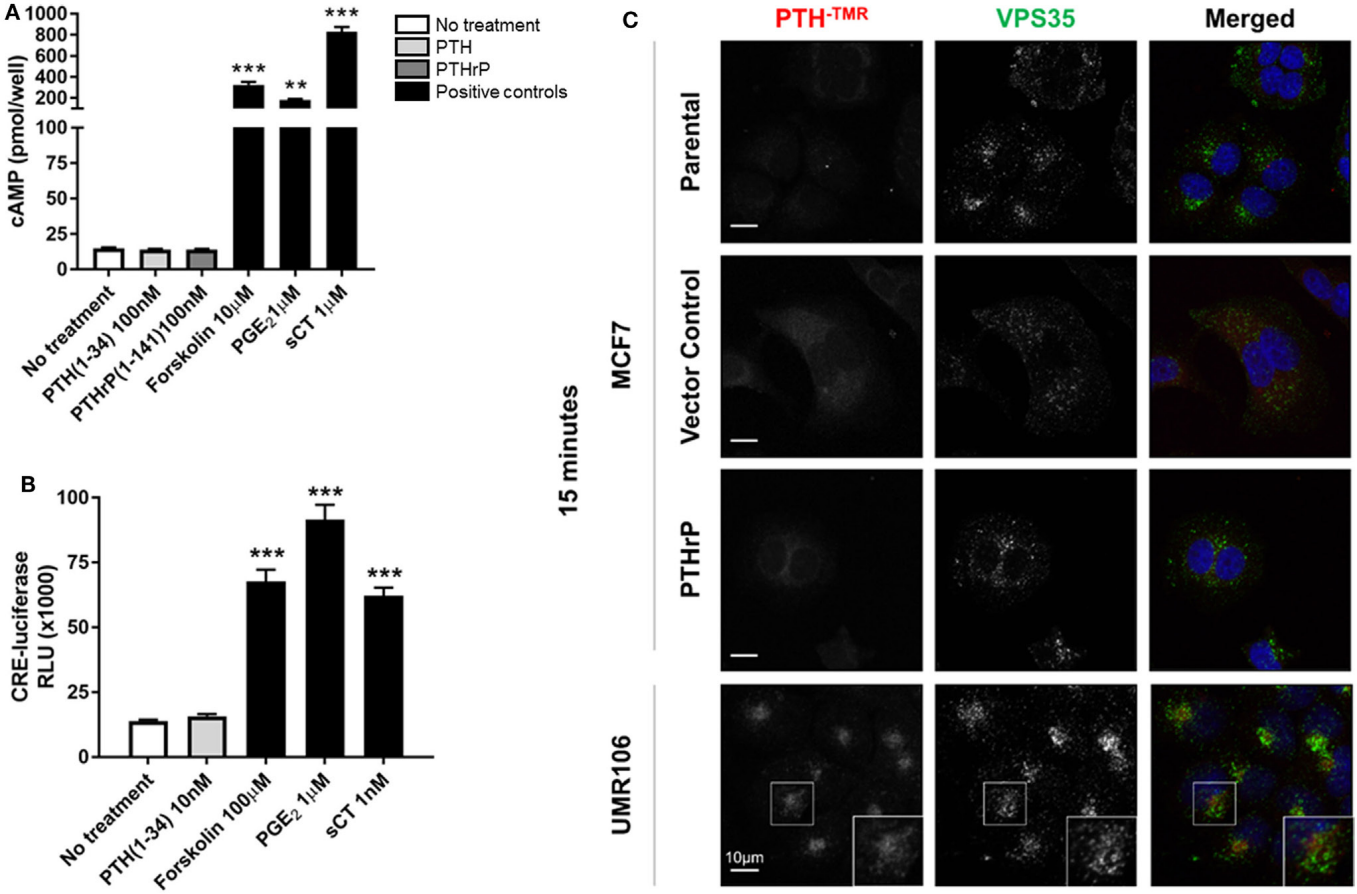
Neither parathyroid hormone (PTH) nor parathyroid hormone-related protein (PTHrP) bind to/activate cyclic AMP (cAMP) in MCF7 cells. **(A)** cAMP production in MCF7 cells following 12 min stimulation with PTH(1–34) or PTHrP(1–141), or positive controls forskolin, prostaglandin E_2_ (PGE_2_), or salmon calcitonin (sCT). Graphs = mean + SE. *n* = 3 replicates from independent experiments. **p < 0.01, ***p < 0.001 vs no treatment by one-way ANOVA with multiple comparisons. **(B)** cAMP response element (CRE)-luciferase signal following 4 h stimulation with PTH or positive controls forskolin, prostaglandin E_2_ (PGE_2_), or sCT. Graphs = mean + SE. *n* = 3 replicates from independent experiments. ***p < 0.001 vs no treatment by one-way ANOVA with multiple comparisons. **(C)** Confocal images of stable MCF7 and UMR106-01 cells cultured on poly-l-lysine-coated glass coverslips and serum starved for 1 h prior to the addition of tetramethylrhodamine-labeled PTH(1–34) (PTH^-TMR^, 100 nM) for 15 min at 37°C. Cells were fixed in 4% PFA and immunostained for the endogenous retromer subunit, vacuolar protein sorting 35 (VPS35). Scale bar, 10 µm. Representative of *n* = 3 independent experiments.

MCF7 cells robustly induced cAMP formation in response to forskolin, PGE_2_, and sCT, but treatment with high dose PTH(1–34) or PTHrP(1–141) elicited no cAMP response (Figure 2A). This confirmed the lack of a cAMP response to PTH in MCF7 cells as reported at the time of discovery of the functional calcitonin receptor (15). In order to investigate later cellular responses, MCF7 cells were transiently transfected with a cAMP response element (CRE)-luciferase construct (CRE-Luc). Treatment with either sCT or PGE_2_ resulted in substantial activation of the CRE-Luc reporter, with no detectable effect of PTH(1–34). All were used at multiple doses in repeated experiments, with no measureable effects detected (Figure 2B).

Tetramethylrhodamine-labeled PTH (PTH^-TMR^) has proven useful for monitoring the surface binding and internalization of amino-terminal PTH upon its target cells through the PTHR1 (23). Vacuolar protein sorting 35 (VPS35) is an essential subunit of the mammalian retromer trafficking complex, where retromer coordinates both retrograde (endosome-to-Golgi) and recycling (endosome-to-plasma membrane) of many cell surface receptors (28), including PTHR1 (23, 29) along the endocytic pathway. VPS35, therefore, serves as a marker of internalized PTH^-TMR^–PTHR1 ligand-receptor complexes following their sequestration into early endosomes (23). Accordingly, the addition of PTH^-TMR^ at saturating conditions (100 nM) for 15 min to UMR106-01 cells, was sufficient to visualize encapsulated ligand–receptor complexes in early endosomes, as determined by its co-localization with VPS35 (Figure 2C). This event coincides with the generation of cAMP following stimulation with either PTH and PTHrP peptides with identical dose responses (19). In contrast, neither PTH^-TMR^ internalization nor co-localization with VPS35 was detected in MCF7 parental, vector-transfected, or PTHrP-transfected cells (Figure 2C).

### Lack of cAMP Gene Response in MCF7 Cells

**Figure 3:**
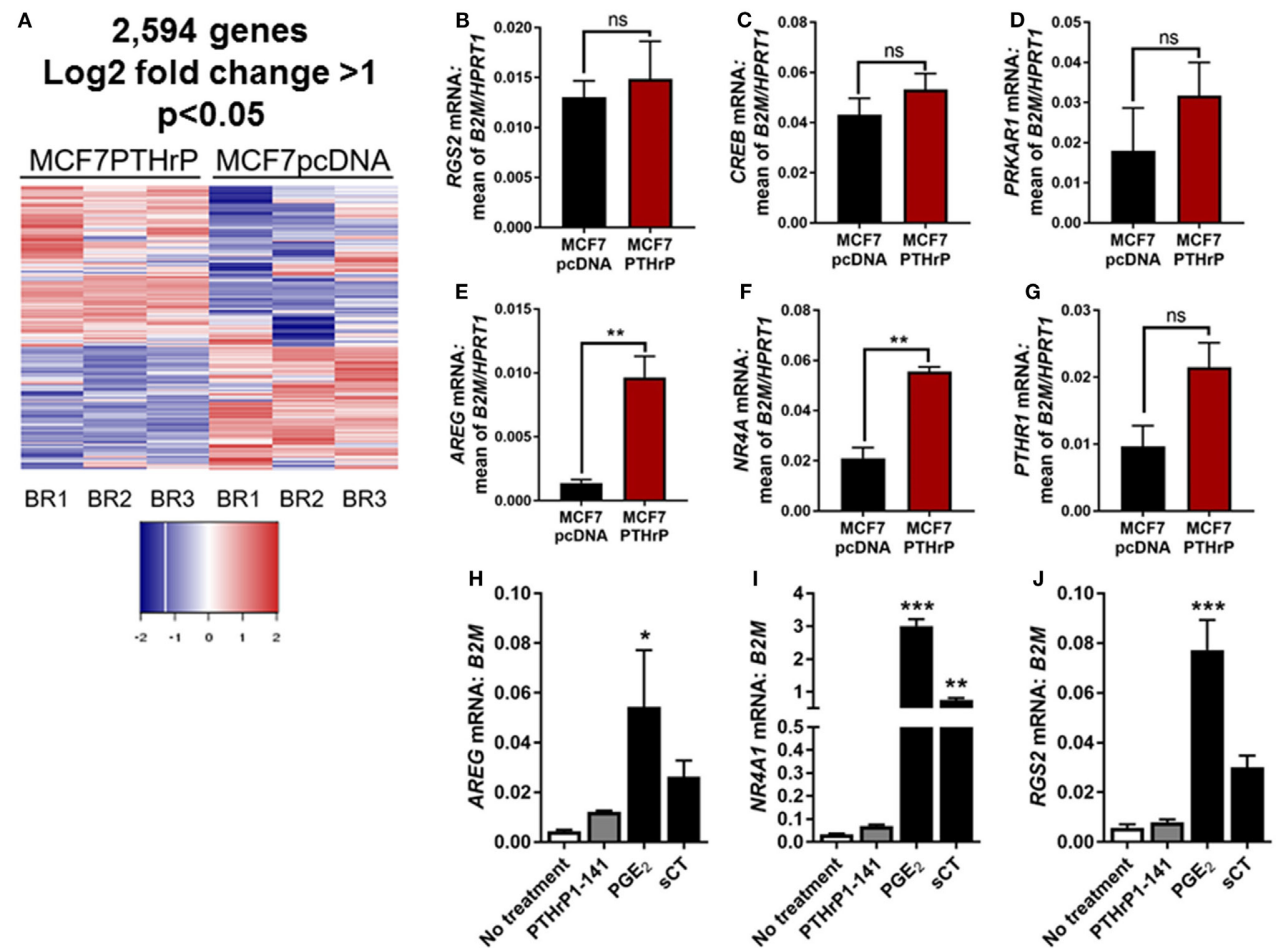
Parathyroid hormone-related protein (PTHrP) overexpression does not induce cyclic AMP (cAMP) target genes. **(A)** Heat map of gene expression with 95% confidence intervals in MCF7pcDNA (empty vector control) or MCF7 PTHrP-overexpressing cells. BR1 = biological replicate 1, BR2 = biological replicate 2, BR3 = biological replicate 3. **(B–G)** qPCR for cAMP target genes in MCF7pcDNA or MCF7 PTHrP-overexpressing cells. mRNA levels were normalized to the geometric mean of *B2M* and *HPRT1* housekeeping genes. Graphs = mean + SE. **p < 0.01 by unpaired Student’s *T*-test. **(H–J)** qPCR for cAMP target genes in MCF7 cells following stimulation with PTHrP(1–141) or positive controls prostaglandin E_2_ (PGE_2_) or salmon calcitonin (sCT). Graphs = mean + SE. *n* = 3 replicates from independent experiments. *p < 0.05, **p < 0.01, ***p < 0.001 vs no treatment by one-way ANOVA with multiple comparisons.

In order to identify novel dormancy genes regulated by PTHrP, we used RNAseq to analyze which pathways are activated in response to PTHrP overexpression in MCF7 cells. We identified >2,500 genes differentially regulated with a log_2_ fold change >1 and *p* < 0.05 in MCF7 PTHrP-overexpressing vs MCF7 control cells (Figure 3A). Consistent with our finding that neither PTH nor PTHrP induce cAMP formation or early post-receptor activation events in MCF7 cells, RNAseq analysis confirmed that only 2 of a previously described panel of 32 CREB-responsive genes (22) were significantly upregulated in MCF7 PTHrP-overexpressing cells (Table 1). Three CREB-responsive genes were significantly downregulated, and the remaining 27 were not altered by PTHrP over-expression, confirming that even long term overexpression of PTHrP does not induce genes that result from cAMP signaling in MCF7 cells.

**Table 1:** Cyclic AMP (cAMP) signaling is not induced by parathyroid hormone-related protein (PTHrP) in MCF7 cells.

Gene name	Log_2_ fold change	*p*-Value	Direction
*AREG*	3.58	1.25E-07	Up
*NRP1*	0.75	5.12E-04	Up
*FOS*	−0.74	0.03	Down
*AQP3*	−1.07	1.14E-03	Down
*CEBPD*	−0.83	0.03	Down
*SOX9*	−0.41	0.41	–
*NR4A3*	−0.07	0.93	–
*BTG2*	−0.29	0.53	–
*UGDH*	0.06	0.87	–
*DUSP1*	−0.12	0.75	–
*NR4A2*	0.14	0.69	–
*GEM*	−0.47	0.66	–
*RGS2*	−0.12	0.93	–
*TCF7*	0.19	0.56	–
*VEGFA*	−0.03	0.96	–
*NR4A1*	0.52	0.53	–
*TEX2*	0.04	0.88	–
*IFNGR1*	0.07	0.88	–
*EFNB2*	0.53	0.10	–
*SIK2*	−0.16	0.49	–
*PLAUR*	0.39	0.34	–
*BMP8A*	0.14	0.82	–
*JUNB*	0.15	0.72	–
*IER3*	0.83	0.17	–
*USP2*	−0.38	0.40	–
*NFIL3*	0.02	0.95	–
*NFKB2*	0.08	0.75	–
*DLEC1*	0.25	0.65	–
*FOXC2*	0.67	0.65	–
*LST1*	−0.05	0.97	–
*KCNE4*	0.25	0.56	–
*IL6*	0.60	0.81	–
*PTHR1*	0.01	0.99	–

*RNAseq values for 32 known cAMP target genes (22) and *PTHR1* (bottom of table) in MCF7 PTHrP-overexpressing cells compared to MCF7 vector controls*.

*Red = significantly up-regulated, green = significantly down-regulated, gray = no significant change*.

Validation of several candidate CREB-responsive genes in MCF7 PTHrP-overexpressing cell lines maintained at a separate institution was consistent with our RNAseq findings (Figures 3B–E). The one exception was *NR4A1*, which was found to be unaltered by RNAseq, but was significantly upregulated in PTHrP-overexpressing cells by real-time PCR (Figure 3F). We also confirmed that *PTHR1* is not downregulated with PTHrP overexpression (Figure 3G). In addition, treatment with positive controls PGE_2_ and sCT induced significantly greater mRNA levels of CREB-responsive genes *AREG, NR4A1*, or *RGS2*, but exogenous treatment with PTHrP(1–141) had no significant effect (Figures 3H–J).

### RNAseq Confirms PTHrP Overexpression Reduces Pro-Dormancy Genes

We previously reported that PTHrP overexpression in MCF7 cells significantly reduced the pro-dormancy genes *LIFR, SOCS3, TPM1, AMOT, P4HA1, HIST1H2BK, SELENBP1*, and *QSOX1* (9). RNAseq analysis confirmed that 6/8 of these genes were downregulated in MCF7 PTHrP-overexpressing cells (Table 2).

**Table 2:** Dormancy genes are downregulated by parathyroid hormone-related protein (PTHrP) in MCF7 cells.

Gene name	Log_2_ fold change	*p*-Value
*LIFR*	−0.57	*p* = 0.09
SOCS3	−1.18	*p* = 0.01*
*AMOT*	−0.45	*p* = 0.04*
*P4HA1*	−0.54	*p* = 0.02*
*HIST1H2BK*	−0.61	*p* = 0.003**
*SELENBP1*	−0.65	*p* = 2.92 × 10^−5^****
*TPM1*	0.02	*p* = 0.945
QSOX1	−0.35	*p* = 0.13

*RNAseq values for eight pro-dormancy genes (9) in MCF7 PTHrP-overexpressing cells compared to MCF7 vector controls*.

*Green = significantly down-regulated, gray = no significant change*.

*p < 0.05, **p < 0.01, ****p < 0.0001.

### Calcium-Related Pathways Are Activated in Response to PTHrP Overexpression in MCF7 Cells

**Figure 4:**
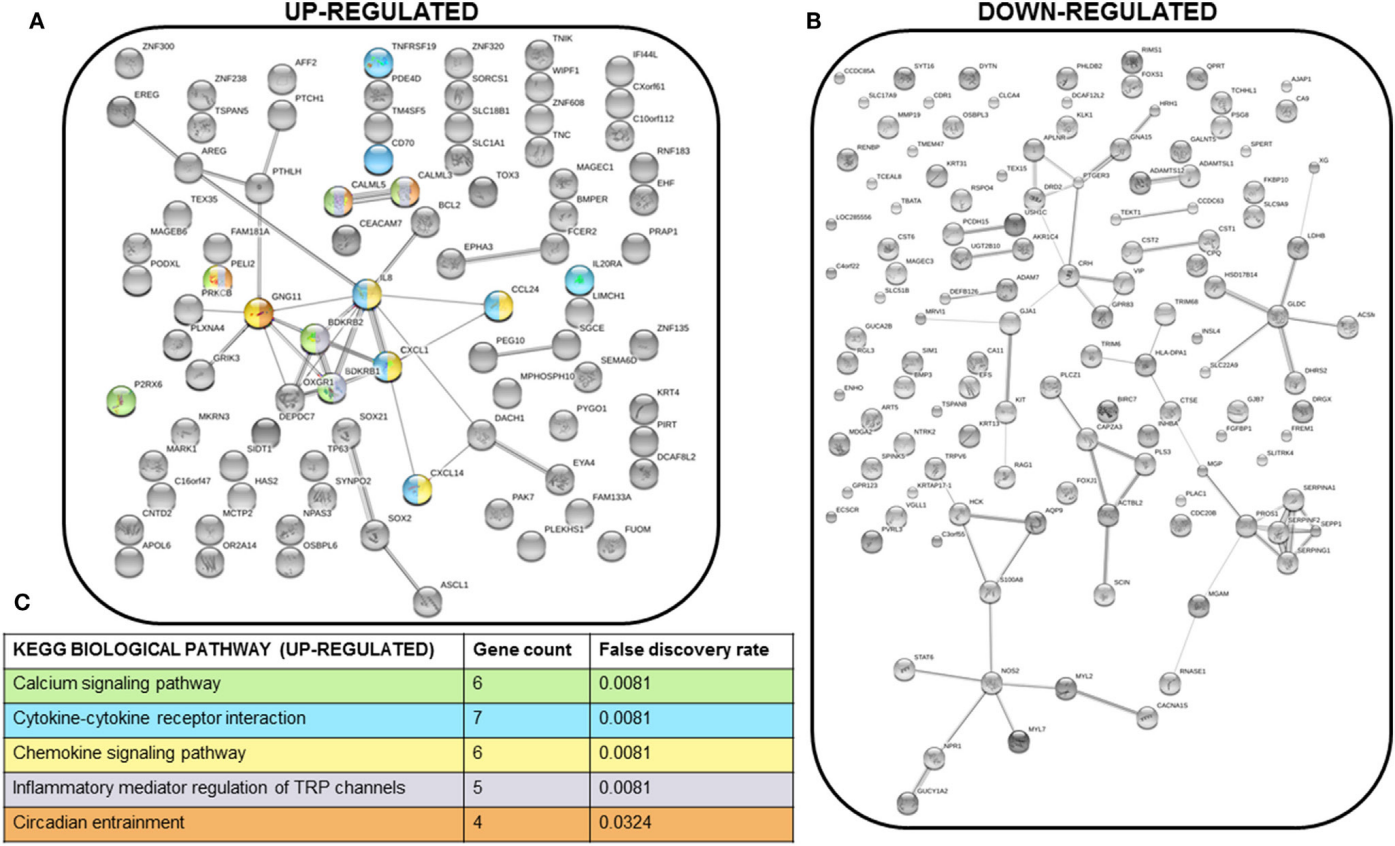
Multiple signaling pathways are upregulated in MCF7 parathyroid hormone-related protein-overexpressing cells. **(A)** STRING network analysis of the top 250 upregulated genes (with log_2_ fold change >1 and *p* < 0.05). Colors of each node correspond to the KEGG pathway indicated in **(C)**. **(B)** STRING network analysis of the top 250 downregulated genes (with log_2_ fold change <−1 and *p* < 0.05). **(C)** KEGG biological pathways significantly enriched for in the STRING analysis of the top 250 upregulated genes. There were no significantly enriched KEGG biological pathways for the top 250 downregulated genes. Colors correspond to the nodes in **(A)**.

We next performed STRING analysis on the RNAseq data to identify significantly enriched pathways. We separately analyzed the 250 upregulated and downregulated genes (all *p* < 0.05) with the largest log_2_ fold change, a total of 500 genes analyzed (Figures 4A,B). STRING pathway analysis of this RNAseq data revealed that the most significantly enriched pathways (false discovery rate = 0.0081–0.0324) in MCF7 cells overexpressing PTHrP in comparison to parental MCF7 cells and across all 500 genes, were the calcium signaling pathway, cytokine–cytokine receptor interaction, chemokine signaling pathway, and inflammatory mediator regulation of transient receptor potential (TRP) channels (Figure 4C).

**Figure 5:**
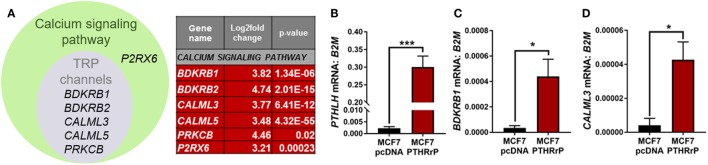
The calcium signaling pathway is significantly enriched downstream of parathyroid hormone-related protein (PTHrP) in MCF7 cells. **(A)** Venn diagram indicating the calcium signaling pathway and transient receptor potential (TRP) channel genes that were significantly upregulated in PTHrP-overexpressing cells (gray circle). There was also one significantly enriched gene that was unique to the calcium signaling pathway, P2RX6 (green circle). **(B)** *PTHLH* mRNA levels, shown as a control for PTHrP overexpression, in MCF7pcDNA control or MCF7 PTHrP-overexpressing cells **(C,D)**. qPCR for mRNA levels of calcium signaling pathway genes in MCF7pcDNA or MCF7 PTHrP-overexpressing cells. Graphs = mean + SE. *n* = 3 replicates from independent experiments. *p < 0.05, ***p < 0.001 by Unpaired Student’s *T*-test.

The calcium signaling pathway and TRP channels are ion channels with high selectivity for Ca^2+^ (30), indicating calcium signaling is dramatically altered with PTHrP overexpression. There was overlap of 5/6 regulated genes in the “calcium signaling pathway” and “regulation of TRP channel pathway” from STRING analysis (*P2RX6* was specific for the calcium signaling pathway) (Figure 5A); there were no unique TRP channel pathway genes that were regulated. mRNA levels for *PTHLH* (control), *BDKRB1*, and *CALML3* (Figures 5B–D) confirmed the RNAseq findings in MCF7 PTHrP-overexpressing cells.

## Discussion

This work provides extensive evidence that PTHrP, although it is capable of inducing substantial changes in gene expression and behavior in MCF7 cells, does not signal through the PTHR1 to activate the cAMP pathway in these cells. Although PTHR1 is detected by qPCR, no cAMP response was detected, and no activity was observed in a CREB reporter assay. Furthermore, out of all the known cAMP responsive genes, only 2 of 32 were regulated in a positive direction by RNAseq analysis. In contrast, PTHrP overexpression in these cells upregulated genes associated with the calcium signaling pathway.

When human breast cancer cells were found to express functional receptors for calcitonin and PGE_2_ linked to adenylyl cyclase activation, no such activation could be detected in response to PTH(1–34) (15). We confirm this observation in the present experiments and show that PTHrP(1–141) also lacks this activity. In addition, we report that PTH(1–34) has no effect on activation of a CREB reporter construct that is readily activated by either sCT or PGE_2_. The latter two agonists, unlike PTH and PTHrP, also promoted expression of genes known to be regulated by the PKA–CREB pathway. There were only two cAMP responsive genes that were significantly upregulated with PTHrP overexpression by RNAseq: *AREG* and *NRP1*. Both of these genes have been implicated in cancer. *AREG* is essential for estrogen receptor-targeted therapeutic response (31). *NRP1* has been previously shown to promote tumorigenesis by enhancing angiogenesis (32) and NRP1-positive cells have been reported to have tumor-initiating properties (33). Thus the upregulation of these genes may result from indirect effects independent of cAMP, a possibility we will investigate. It is also worth noting that the PTHrP induction of *AREG* mRNA, and the CREB-responsive gene *NR4A1*, in MCF7s is much lower than its induction with the positive controls prostaglandin E_2_ (PGE_2_) and sCT. In a separate study, we have tested the same secreted form of PTHrP, and the same preparation of recombinant PTHrP(1–141) in Ocy454 cells, an osteocyte cell line that expresses the PTHR1 (7). Overexpression and exogenous treatment both induced a significant increase in cAMP in these cells, and overexpression increased the CREB responsive genes, *Nr4a1* and *Rgs2* (7) confirming that these forms of PTHrP are capable of inducing a CREB response, but not in MCF7 cells.

Our data also indicate that PTH, which shares with PTHrP the same ability to bind to the PTHR1, does not bind to MCF7 cells in any detectable manner. This is illustrated by use of the PTH^-TMR^ reagent, which requires functional PTHR1 for CREB activation and internalization into early endosomes. This suggests that the action of overexpressed PTHrP that suppresses dormancy and results in major changes in gene expression and osteolytic destruction of bone, is not only not cAMP-mediated, but is also not elicited through the PTHR1. However, we have not excluded the possibility that PTHrP binds to PTHR1 at levels below our detection limits, and initiates cAMP-independent signaling.

Parathyroid hormone and PTHrP have identical amino acids in 8 of their first 13 residues, but other similarities within the sequences are no more than would be expected by chance (1, 3). In PTHR1-bearing target cells, recombinant PTHrP(1–141) and synthetic shorter amino-terminal forms were equipotent on a molar basis with each other and with PTH(1–34) in their ability to promote cAMP activity (19). In exerting this function, PTHrP and PTH were shown to share actions upon a common receptor, PTHR1 (14). These functions are absent in MCF7 cells. Instead, our findings suggest that the major changes in gene expression in MCF7 cells in response to PTHrP must occur through PTHrP actions mediated by domains of PTHrP distinct from the (1–34) region known to act on the cAMP–PKA pathway through PTHR1.

A number of biological activities have been ascribed to domains of PTHrP beyond the amino-terminal region, but although these domains have been defined on the basis of the primary amino acid sequence, no receptors for these responses have yet been identified. For example, (i) the mid-molecule portion, between residues 35 and 84, is responsible for placental calcium transport, (ii) many pharmacologic studies have shown biological effects of the C-terminal domain, beginning at residue 107, and (iii) a nuclear localizing sequence mediates transport of PTHrP to the nucleus in many cell types [reviewed in Ref. (1)]. These many biological activities within the PTHrP molecule led to it being regarded as a multifunctional cytokine (34, 35) but the specific intracellular pathways that mediate these non-PTHR1-mediated actions remain unknown. These possibilities have been raised recently with respect to the role of PTHrP in bone remodeling (7), since mice lacking PTHrP in osteocytes exhibit a bone phenotype that is markedly different from mice lacking the PTHR1 (36).

Thus in this work, where substantial effects of PTHrP overexpression on gene expression in MCF7 cells seem to be unrelated to PTHR1-mediated actions though cAMP/PKA/CREB activation, these other domains of PTHrP need to be considered. This question begins to be addressed with the findings from the RNAseq data, which identified the calcium signaling pathway as significantly upregulated by PTHrP overexpression. In cancer, upregulation of Ca^2+^ channels and pumps promotes tumor proliferation and drives tumorigenesis. Several of these signaling pathway components have been reported as overexpressed in breast, prostate, colon, pancreas, and lung tumors (37–39). It has also been shown that PTHrP nuclear action downstream of the calcium-sensing receptor (CaSR) promotes proliferation and reduces p27^Kip1^ levels in breast cancer cells, ultimately preventing nuclear accumulation of apoptosis-inducing factor and the cell death that normally occurs when Ca^2+^ levels are in excess (40). While these actions have never been directly linked to PTHrP-induced bone destruction, our findings are consistent with the known roles for the calcium signaling pathway in cancer. These data suggest that CaSR acts upstream of PTHrP, and our data raise the possibility that PTHrP further promotes calcium signaling, possibly in a feed-forward loop.

We previously reported that PTHrP overexpression in MCF7 cells downregulates eight pro-dormancy genes (9) and our RNAseq analysis now provides a potential pathway through which PTHrP may function to downregulate these genes. Experiments to determine the functional significance of the calcium signaling pathway in tumor dormancy *in vivo* will be necessary to determine whether this is the pathway through which PTHrP enables dormant tumor cells to aggressively colonize the bone.

## Author Contributions

RJ, YS, PH, AC, and JJ performed experiments and analyzed data. RJ, NP, NS, and TM interpreted the data. RJ, NS, and TM wrote the manuscript. YS, PH, AC, JJ, and NP edited the manuscript.

## Conflict of Interest Statement

The authors declare that the research was conducted in the absence of any commercial or financial relationships that could be construed as a potential conflict of interest.
